# Novel modeling methodologies for the neuropathological dimensions of Parkinson's disease

**DOI:** 10.3934/Neuroscience.2020006

**Published:** 2020-04-10

**Authors:** Panagiotis Vlamos

**Affiliations:** Department of Informatics - Ionian University, Corfu, Greece

Parkinson's disease (PD) is the second most common neurodegenerative disorder that mainly affects the dopaminergic neurons in the area of the brain that controls balance and movement, known as substantia nigra. Dopaminergic neurons secret dopamine, a key messenger molecule that communicates movement signals to other parts of the brain. When these neurons start to atrophy or die, the dopamine levels decrease. Thus, the body becomes unable to control motor movements and the PD symptomology begins. Although the exact cause of the disease is still unknown, aging is believed to be a very common risk factor. PD pathogenesis involves genetic and environmental factors, oxidative stress, mitochondrial dysfunction, and dysfunction of ubiquitin-proteasome system [1]. There are two types of dememntia that affect PD patients: 1. Parkinson's dementia and 2. dementia with Lewy bodies (LBs). Parkinson's disease is also characterised as a proteinopathy. The proteins involved in PD include a-synuclein and parkin. The histological hallmark of PD is the presence of fibrillar aggregates known as Lewy bodies (LBs) [1]. Their formation is considered a marker of neurodegeneration. Studies have shown that a-synuclein is a major constituent of LB fibrils, since: (i) significant loss of neurons is found in the predilection sites for LBs, predominantly in the substantia nigra and locus ceruleus, (ii) the number of LBs in patients with mild to moderate loss of neurons in the substantia nigra is higher compared to patients with severe neuronal depletion, suggesting that neurons which contain LBs may be dying neurons and (iii) cortical LB density could be one of the major correlates of cognitive impairment in PD and Lewy Body Dementia. Reports suggested that protofibrils and oligomers of a-synuclein are cytotoxic, and that fibrillar aggregates of a-synuclein may possess a cytoprotective effect regarding PD.

Early detection of neurodegenerative diseases is crucial, as it can give the opportunity to the patient to take part in an appropriate and effective therapeutic protocol. Early diagnosis leads to better treatment and management of the disease. Because of the difficulties faced when diagnosing a neurodegenerative disease, medical research seeks effective non-invasive diagnostic molecules to be used for early detection of the appearance of PD symptoms, at a time when pharmacological interventions are still possible. Computational tools such as machine learning (ML) techniques have a variety of applications in bioinformatics such as in biomarker discovery. Biomarkers are most commonly utilized for disease diagnosis and progression as well as disease prediction. Biomarkers allow the physician to adapt the treatment to each particular patient, since they will be able to monitor disease progression. Biomarkers can be broadly classified as: i) biological, ii) digital and iii) computational.

Biological biomarkers are characteristics that can be objectively measured and evaluated as indicators of a physiological as well as a pathological process or pharmacological response to a therapeutic intervention. Digital biomarkers are defined as objective, quantifiable physiological and behavioral data that are collected and measured by means of digital devices such as such as portables, wearables, implantables or digestibles. The data collected is typically used to explain, influence and/or predict health-related outcomes. Once said data are directly linked with a health-related outcome, they are then defined as digital biomarkers. According to Aitken, et al. [2] approximately 4300,000 unique apps are on the market powering close to 350 different types of consumer-worn devices. Computational biomarkers include both biological and digital biomarkers. The need of specialised computational tools emerges for the study of biomarkers, due to their complex nature. Data mining aims to extract finding/outcomes that can't be obtain through traditional computational tools. Mathematical models can correlate biomarkers, resulting in the formation of decision trees that demonstrate the significance of each biomarker. Decision trees are made of leaves and branches which respectively represent classifications and combinations of traits that lead to said classifications. An ensuing goal is not the discovery of novel biomarkers but their correlation with pharmaceuticals and therapeutic intervention.

Predictive models for biomarker discovery include the selection of a supervised learning algorithm according to the characteristics of the multivariate data and the objective of the research. Predictive models aim to correctly classify new unlabeled data into healthy or PD. The framework for the process of the biomarker discovery process, includes: 1. feature identification, 2. model selection (involves the identification and utilization of the most efficient ML technique) and 3. performance analysis [3].

**Figure 1.**
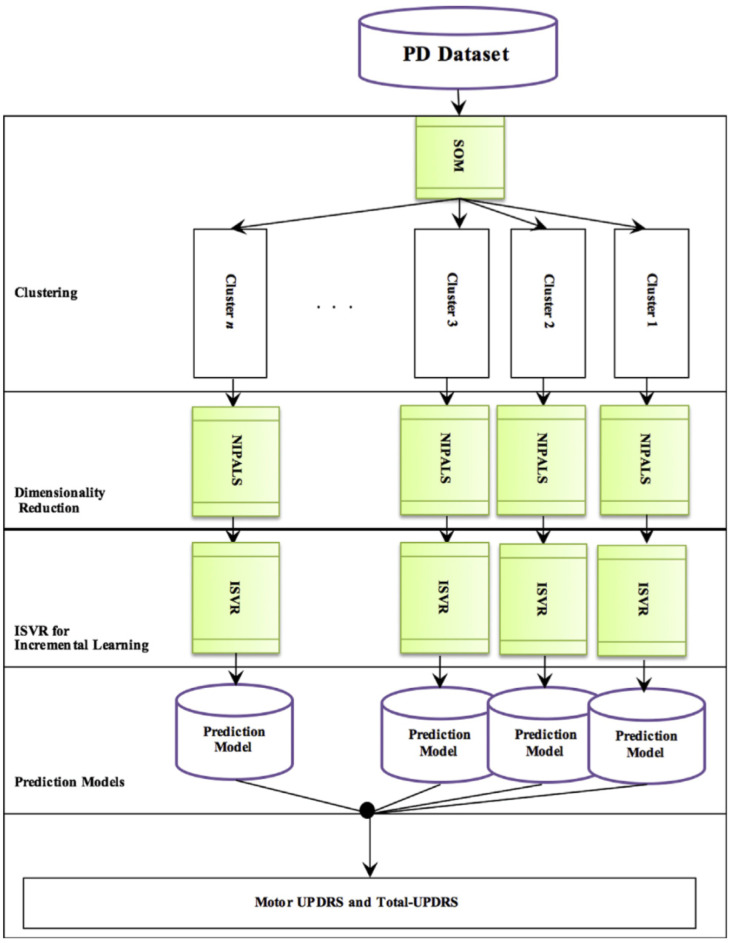
Machine learning techniques utilized for the creation of a PD prediction model [4].

Taking into consideration that time of calculation and accuracy are two important criteria for the evaluation of medical diagnostic systems [4] proposed a new model using Incremental Support Vector Support Regression (ISVR), an effective regression engineering technique. As can be seen in figure 1, the first step of the research methodology for this model involves data clustering using SOM (self-organizing map). In the second step, NIPALS (Nonlinear Iterative vartial Least Squares algorithm) was utilized for data dimensionality reduction. In the last step, the ISVR technique was used for the prediction of Total-UPDRS (Unified Parkinson's Disease Rating Scale) and Motor-UPDRS observed in the data.

Additional modelling methodologies in PD include advanced experimental in vitro models that examine disease mechanisms as well as other aspects of this disorder. Smits, et al. [5] created a robust method to reproducibly generate 3D human midbrain organoids containing midbrain dopaminergic neurons to investigate disease (PD-related) mechanisms and pathophysiology.

Other novel modelling methodologies of PD include animal models. C. elegans is a common model of PD, since it offers several advantages such as easy genetic manipulation, rapid and low-cost experiments, and large-scale screenings for disease modifiers. A number of C. elegans models regarding PD have been produced that have exhibited phenotypes that allow for the observation of disease mechanisms and identification of therapeutic targets [6].

However, the development of effective animal models of PD is not easily accomplished. Induced pluripotent stem cells (iPSCs) allow for the generation of patient-specific dopaminergic neurons to study PD. In combination with the ability of genome editing, isogenic iPSCs can be generated and aid in the analysis of patient-specific midbrain dopaminergic neurons. Thus, providing a valuable tool for the examination of PD-mechanisms and drug discovery [7].

Bakshi, et al. [8] proposed an α-synuclein (Asyn) aggregation model for the discovery of PD-related therapeutic agents and the better understanding of disease mechanisms. This model incorporated feedback among Asyn, dopaminergic neurons as well as mitochondria.

There is a vast body of research that proposes models of PD pathophysiology and pathogenesis, by incorporating interdisciplinary approaches. Like it was mentioned above, the exact disease mechanisms of PD have not been clearly indicated yet. Therefore, models have been created in order to not only gain a better insight into PD's mechanism but also possibly identify effective drug targets.
